# Predicting factors of organizational citizenship behavior in Indonesian nurses

**DOI:** 10.1016/j.heliyon.2021.e08652

**Published:** 2021-12-21

**Authors:** Idris, Nanang AS, Budi Eko Soetjipto, Achmad Sani Supriyanto

**Affiliations:** a Universitas Negeri Malang, Malang, Indonesia; b Universitas Brawijaya, Malang, Indonesia; c Universitas Islam Negeri Maulana Malik Ibrahim, Malang, Indonesia

**Keywords:** Job satisfaction, Organizational citizenship behavior, Learning culture, Nurses, Transformational leadership

## Abstract

This present study investigates the relationship between transformational leadership, learning culture, job satisfaction, and organizational citizenship behavior. In addition, it also examines the mediating role of learning culture and job satisfaction on transformational leadership and organizational citizenship behavior. This study utilized online questionnaires to collect data from nurses in a public hospital in Indonesia. Partial least square (PLS) was used as an analysis tool for 205 collected data. The findings indicated that transformational leadership did not significantly affect organizational citizenship behavior, but this has substantial implications for learning culture and job satisfaction. Both learning culture and job satisfaction significantly and positively predict organizational citizenship behavior. In addition, learning culture and job satisfaction act as perfect mediators between transformational leadership and organizational citizenship behavior. This study suggests leaders to maximize a systematic learning program and to pay attention to the nurses’ job satisfaction rate to improve organizational citizenship behavior. The findings also provide learning culture and job satisfaction as critical factors, directly and indirectly, in motivating employees to perform organizational citizenship behavior.

## 1 Introduction

Employees' extra-role behavior or commonly known as organizational citizenship behavior (OCB), is indispensable for current organizations, especially in the context of hospitals in Indonesia which recently experienced an increase in the number of patients due to the high transmission of the Corona Virus-19 (Gusrita and Syahrizal, 2020). This condition can help employees complete their tasks effectively through voluntary helpful behavior (Mekpor and Dartey-Baah, 2017). OCB is multidimensional employee behavior that includes various aspects of positive employee behavior and is beneficial for organizational development (Khalili, 2017; Sani et al., 2018) and can reduce turnover (Saoula et al., 2018). OCB becomes more interesting in social relations because it elevates the status of those who participate in it as colleagues or friends (Jo and Joo, 2011). This behavior also increases employee commitment so that hospitals can maintain their sustainability (Zurahmi et al., 2019).

OCB behavior becomes an essential aspect for leaders in encouraging their subordinates to behave beyond the formal requirements of their work. Leaders have been identified as having a crucial role in influencing the behavior of their subordinates (Bass et al., 2008; Idris et al., 2020b). Leadership as a skill in influencing others is a vital element for changing the behavior of individuals and directing them to achieve organizational goals (Robbins and Judge, 2017). Good leadership is defined as those who can inspire their members to work harder and conduct better than they did previously (Mekpor and Dartey-Baah, 2017). Bass et al. (2007) mentioned this as transformational leadership (TL). As a leadership trait, motivating and promoting rational thinking have significant implications for employees' innovative behavior, resulting in increased cooperation (Hambali and Idris, 2020; Idris and Adi, 2019). Supriyanto et al. (2020) stated that TL had a substantial role in predicting OCB. Nurses with high OCB are closely related to the role of an inspiring leader (Jun, 2017).

Concerning encouraging OCB among nurses, building a learning culture (LC) also needs more attention from leaders and organizations. This occurs at the level of individuals, teams, and organizations as a whole which is at least characterized by the process of acquiring and sharing new knowledge (Garvin, 1993). Organizations that facilitate learning among their employees impact their desire to reciprocate by displaying OCB (Eisenberg et al., 2018; Danish et al., 2014). Furthermore, previous studies have also stated that job satisfaction (JS) convincingly promotes OCB in health professionals (Ng et al., 2019). JS describes how well the individual's overall perception of his job (Idris et al., 2020a; Luthans, 2011). Empirical studies prove that JS has a strong relationship with various positive work outcomes of nurses, such as in-role and extra-role performance (Biagioli et al., 2018) and teamwork collaboration (Monroe et al., 2021).

Although several previous studies have investigated the OCB of health professionals in the hospitals (Ng et al., 2019; Biagioli et al., 2018), there are still inconsistencies in the findings, especially regarding the relationship between TL and OCB. Kim (2014) and Arifiani et al. (2016) found that TL did not significantly encourage nurses' OCB. This is contrary to what has been previously mentioned: there is a strong correlation between the two variables (Jun, 2017; Supriyanto et al., 2020). In addition, LC has been shown to strongly contribute to increasing OCB (Islam et al., 2016). Nurses' OCB also increased when employees are satisfied with their work (Biagioli et al., 2018). Therefore, we propose LC and JS as TL mediators in predicting OCB. These findings have emerged to the significant investigation whether this proposed framework acceptable or not. This fills the research gaps, empirically, regarding the inconsistency of findings and predictors of TL and OCB, particularly in public hospital, and theoretically, regarding the proposed framework by constructing LC and JS as mediators between TL and OCB. Thus, this study investigates the relationship between TL and OCB by exploring LC and JS, which have great potential as mediators.

## 2 Literature review and hypotheses development

### 2.1 Transformational leadership and organizational citizenship behavior

Scholars in the field of organizational behavior in the 1960s began to investigate the leader-follower relationship and attempted to classify behavior and what causes it. There were also investigations into employee behavior, such as discretionary behavior, which received more attention from academics (Ocampo et al., 2018). Katz (1964) was the first to observe behavior that went beyond formal duties in the workplace. According to Katz and Kahn (1966), every organization requires discretionary behavior that accommodates the needs. Thus, organizational members who engage in these behaviors contribute to organizational effectiveness. Although Katz (1964) and Katz and Kahn (1966) was the first scholar to investigate discretionary behavior in the workplace, Smith et al. (1983) was the first to coin the term organizational citizenship behavior (OCB). According to Organ (2016), behavior that can be classified as OCB has certain features. First, the behavior must be within the control of the individual, where he has the freedom to choose and to show the behavior or not. Second, this behavior is not recorded in the organizational system. Third, although only slightly, this behavior contributes to improving the functioning of the organization.

Leadership, including TL, has a vital role in changing employees’ behavior (Nguni et al., 2006). TL is defined as a skill in motivating and inspiring others to be moved and to work beyond the expectation (Bass, 1985). This leadership considers subordinates as employees and whole persons, which profoundly impact employees (Robbins and Judge, 2017). Bass et al. (2008) defines a leader with a transformational model as a person with three main components, including the ability to: (1) push its members to a higher level of awareness of the urgency and value of their work and how to achieve these results; (2) get followers to work beyond their interests to sustain organizational and team functioning; and (3) encourage the needs of its members from lower levels to higher needs in the form of achievement and self-actualization.

Empirically, the study conducted by Jun (2017) involving 219 nurses demonstrated that TL has an influential role in fostering nurses' extra-role behaviors. Similarly, Zurahmi et al. (2019) also showed that the OCB of employees in hospitals is strongly related to the role of TL. TL has significant implications in building employee OCB (Supriyanto et al., 2020; Mekpor and Dartey-Baah, 2017). Therefore, we formulated the first hypothesis regarding the relationship between TL and OCB.

H1 There is a significant positive effect of TL on OCB.

### 2.2 Learning culture and organizational citizenship behavior

In terms of organizational learning, learning organization has been used interchangeably in various literature, which has led to confusion in using these terms. Popper and Lipshitz (2000) defined organizational learning as a process that creates continuous knowledge transfer among employees and integrates learning into corporate systems to reach strategic objectives. The definition of a learning organization is conveyed by Robbins and Judge (2017) that a learning organization is an organization that has developed a sustainable capacity to adapt and change. Meanwhile, learning culture (LC) is a learning organization concept from the point of view of organizational culture (Egan et al., 2004). This happens at the level of individuals, teams, and organizations that have been systemized (Marsick, 2013), which is at least characterized by the process of acquiring and sharing new insights (Garvin, 1993).

Scholars agree that organizational culture and the learning process shape people's behavior (Champoux, 2011), like employee OCB. A corporate learning culture is a strong predictor of OCB, both individual and organization (Saoula et al., 2018). A study on 452 workers' public organizations in Korea showed that LC is significantly associated with OCB (Jo and Joo, 2011). Likewise, Eisenberg et al. (2018) also researched LC and OCB of employees' public sector. It showed that there was a significant relationship between these variables. Although there have been many studies on the correlation between these two variables, there is a lack of studies on the linkage of LC and OCB, mainly in the hospitals.

H2 There is a significant positive effect of LC on OCB.

### 2.3 Job satisfaction and organizational citizenship behavior

Job satisfaction (JS) refers to an individual's emotional reaction to his work, which is determined by the extent to which the difference between what individuals get from their work and what they expect (Gruneberg, 1979). Luthans (2011) defined JS as a description of the extent to which individuals perceive their work as a whole. Engaging in JS is relevant to improving employee welfare and its positive contribution to organizational productivity (Gruneberg, 1979). On the other hand, dissatisfaction can negatively impact the organization's functioning, such as low employee commitment, poor performance, and high turnover (Na-Nan et al., 2020; Vermeir et al., 2018). Thus, a high JS becomes essential for employees, especially nurses in hospitals, who tend to be prone to fatigue due to the continuous nature of work (Lee et al., 2020).

JS is closely related to employee behavior in the workplace. Empirical evidence shows that satisfied individuals with their jobs tend to have positive behaviors, such as voluntary helping behavior (Narzary and Palo, 2020). JS is a predictor that directly influences the three dimensions of employee OCB: employee compliance, loyalty, and participation (Hurst et al., 2017). Several previous studies have carried out the relationship between JS and OCB nurses (Margerison, 2001; Ng et al., 2019; Biagioli et al., 2018), who revealed that professional health workers display OCB frequently when they are satisfied with their work.

H3 There is a significant positive effect of JS on OCB.

### 2.4 Transformational leadership, learning culture, and organizational citizenship behavior

Leadership has been verified as an essential factor in building organizational culture, and LC (Schein, 2010). Froehlich, Segers and Bossche (2014) validated that leadership style can instil learning in every work activity of organizational members, both formally and informally. Previous studies have also shown that the type of TL enables organizations to learn through experimentation, exploration, communication, and dialogue (García-Morales et al., 2012). Park and Kim (2018) illustrated that TL is positively and significantly associated with organizational learning stimulated by LC.

LC, as already mentioned, has a strong correlation with high employee OCB (Saoula et al., 2018). Jo and Joo (2011) also revealed that employees in the public sector organization show OCB frequently when they are facilitated to learn and explore new knowledge and safeguards. Therefore, we argue that LC can potentially mediate TL and OCB in nurses in the public hospital sector.

H4 There is a significant positive effect of TL on LC

H5 LC can be a mediator of TL and OCB

### 2.5 Transformational leadership, job satisfaction, and organizational citizenship behavior

TL is consistently related to employees' behavior and perceptions about their work, including in the nursing field. Leaders who display a transformational style in hospitals can create a conducive work environment to increase nurses' perceptions of JS (Boamah et al., 2018). According to Ahmad et al. (2013), TL is more accepted in health sector organizations when compared to transactional leadership, which prioritizes the needs of each individual. Empirically, Ohunakin et al. (2019) proved that individual consideration, inspiration, and intellectual stimulation play a substantial role in improving nurses' JS.

Another study showed that nurses with high satisfaction were encouraged to display OCB frequently (Biagioli et al., 2018). Ng et al. (2019) revealed similar findings that OCBs among health professionals are positively connected to job satisfaction in their workplace. In more depth, a study on 211 teachers in Israel conducted by Nasra and Heilbrunn (2016) found that JS is a mediator of TL and OCB. Although the study was not completed in the health organization sector, it is still helpful for researchers to consider placing JS as a mediating variable.

H6 There is a significant positive effect of TL on JS

H7 JS can mediate the relationship of TL and OCB

## 3 Methods

### 3.1 Sample and procedures

Data were collected for more than one month, from April 5, 2021, to May 15, 2021. Initial contact was made by email with the human resource manager of RSUD Koesma in Indonesia explaining the purpose of the study and the research permit. Once approved, online questionnaires were distributed to nurses by google form. From 210 online questionnaires distributed, 205 questionnaires (97.6%) were collected after deleting five questionnaires that could not be processed due to incomplete answers. Simple random sampling was used in this study, and the Slovin formula was applied to the population accounting for 440 nurses who worked for more than one year. The calculation ended up with 210 respondents.

$$n=\frac{N}{1+{\text{Ne}}^{2}}$$

$$n=\frac{440}{1+\left(440\text{ ​x ​}0.05^{2}\right)}$$

$$n=\frac{440}{1+\left(440\text{ ​x ​}0.0025\right)}$$

$$n=\frac{440}{2.1\;}$$

n=209.52

The distribution of the characteristics of respondents can be seen in Table 1.

**Table 1:** Distribution of respondent characteristics.

	Gender	Age	Education	Length of work	Job-status
Male	48				
Female	157				
≤25 years		13			
26–30 years		73			
31–35 years		33			
36–39 years		35			
≥40 years		51			
Associate degree			85		
Bachelor degree			7		
**Professional (Nurse)**			113		
1–5 years				77	
6–10 years				34	
11–15 years				24	
>15 years				70	
Permanent Nurses					107
Temporary Nurses					98

Table 1 shows that 76.6 percent of the respondents are female, and the remaining 23.4 are male participants. The percentage of respondents in the age group of 26–30 years is 35.6 percent. Participants aged 40 years were 24.9 percent, participants aged 36–39 years was 17.1 percent, participants aged 31–35 years had a percentage of 16.1 percent, and participants aged 25 years was 6.3 percent. Based on education, 55.1% of all participants have taken professional nursing education, 41.5% have an associate degree, and 3.4% have a bachelor's degree in nursing. For the length of work, participants who have worked for 1–5 years are 37.6 percent, those who have worked more than 15 years are 34.1 percent, 6–10 years are 16.6 percent, and 11.7 percent for those who have worked for 11–15 years. Finally, the employment status of nurses shows that 52.2 percent of participants are permanent employees and 47.8 percent are temporary employees.

### 3.2 Measures

This research is a correlational study that covers the variables TL, LC, JS, and OCB. The assessment of the instrument's measurement used reliability and validity test that aims to identify the level of consistency and accuracy of the tool used with the provision that the loading factor values are more than 0.6 and the values of average variance extracted (AVE) exceed 0.5 for construct validity. Furthermore, construct reliability is reviewed based on composite reliability (CR) and Cronbach Alpha (CA) value must be above 0.7 and 0.6 (Hair et al., 2020).

Analysis of the Multifactor Leadership Questionnaire (MLQ) developed by Hartog et al. (1997) was adapted to measure the TL. The MLQ scale consists of four elements, including charisma (3 items), inspiration (3 items), intellectual stimulation (3 items), and individual consideration (3 items). Each item on each construct was measured and graded on a 5-point Likert scale from 1 ″strongly agree to 5 ″strongly disagree." One example is "My leader makes me aware of strongly held values, ideals, and aspirations which are shared in common." Concerning the instrument's validity and reliability in this study, Table 2 exhibits that the value of AVE was higher than 0.5, CR and CA values also exceeded the required values (0,961 and 0.955). Thus, it is stated that the tool is consistent and accurate in measuring its construct.

**Table 2:** Value of loading, AVE, composite reliability (CR) and cronbach alpha (CA).

Variables	Indicators	Items	Loading	AVE	CR	CA
Transformational Leadership (TL)	Charisma	TL1	0,802	0,673	0,961	0,955
		TL2	0,777			
		TL3	0,724			
	Inspiration	TL4	0,862			
		TL5	0,871			
		TL6	0,809			
	Intellectual Stimulation	TL7	0,867			
		TL8	0,865			
		TL9	0,734			
	Individualized Consideration	TL10	0,819			
		TL11	0,860			
		TL12	0,834			
Learning Culture (LC)	Continuous Learning	LC1	0,763	0,602	0,962	0,958
		LC2	0,736			
	Inquary and Dialog	LC3	0,795			
		LC4	0,813			
		LC5	0,759			
	Team Learning	LC6	0,807			
		LC7	0,825			
		LC8	0,730			
	Embedded System	LC9	0,824			
		LC10	0,806			
		LC11	0,723			
	Empowerment	LC12	0,718			
		LC13	0,768			
	System Connection	LC14	0,793			
		LC15	0,711			
	Strategic Leadership	LC16	0,820			
		LC17	0,784			
Job Satisfaction (JS)	Intrinsic Domain	JS1	0,748	0,613	0,926	0,909
		JS2	0,780			
		JS3	0,719			
		JS4	0,778			
	Relational Domain	JS5	0,843			
		JS6	0,773			
		JS7	0,860			
		JS8	0,750			
Organizational Citizenship Behavior (OCB)	Helping Behavior	OCB1	0,812	0,605	0,961	0,956
		OCB2	0,706			
	Sportsmanship	OCB3	0,693			
		OCB5	0,810			
	Organizational Loyalty	OCB6	0,651			
		OCB7	0,788			
	Organizational Compliance	OCB8	0,763			
		OCB9	0,827			
	Individual Initiative	OCB10	0,823			
		OCB11	0,854			
		OCB12	0,831			
	Civic Virtue	OCB13	0,702			
		OCB14	0,782			
		OCB15	0,729			
	Self-Development	OCB16	0,776			
		OCB17	0,864			

The LC was measured by using the dimension of learning organization questionnaires (DLOQ) established by Marsick and Watkins (2003), which involves seven aspects such as continuous learning (2 items), inquiry and dialogue (3 items), team learning (3 items) embedded systems (3 items), empowerment (2 items), system connections (2 items) and strategic leadership (2 item). One example includes "In my organization, people openly discuss mistakes to learn from them." A 5-point Likert scale from 1 ″strongly agree to 5 ″strongly disagree" was used to provide a rating for each question item. Table 2 illustrates the value of AVE was 0.602, CR and CA respectively were 0.962 and 0.958. This indicates that the requirements of reliability and validity construct were fulfilled.

JS was measured by adopting the nursing workplace satisfaction questionnaire (NWSQ) developed by Fairbrother et al. (2010), which includes the 'intrinsic' domain of the work they are involved in (4 items), such as "My job is very meaningful for me," and the 'relational' domain of people who are co-workers (4 items); for instance, "I feel that my colleagues like me." Each item on the construct was measured based on a 5-point Likert scale from 1 ″strongly agree" to 5 ″strongly disagree." Concerning the instrument's validity and reliability, it shows that the value of AVE was 0,613, CR and CA respectively were 0,926 and 0,909. Thus, it is stated that the instrument is consistent in measuring its construct.

The OCB instruments combined Podsakoff et al. (1990) and Podsakoff et al. (2000) instruments which cover the seven main dimensions of OCB, including helping behavior (2 items), sportsmanship (2 items), organizational loyalty (2 items), organizational compliance (2 items), individual initiative (3 items), civic virtue (3 items) and self-development (2 items). Each item on each construct was scored on a 5-point Likert scale from 1 ″strongly agree" to 5 ″strongly disagree." One example is "I willingly help others who have work-related problems." Table 2 depicts that the value of AVE was 0.605, CR and CA respectively were 0.961 and 0.956. This means that the instrument used is satisfied.

## 4 Results and discussion

Partial Least Squares (PLS) analysis with the bootstrapping method using 500 sub-samples was employed after the data was collected. This is intended to test the formulated hypothesis. The hypothesis is accepted if the significance level of the relationship between variables is less than 0.05. Table 3 reveals seven test results of the relationship between variables.

**Table 3:** Hypotheses testing in PLS.

Hypotheses	Relationships	(β)	SE	Sig.	Decision
1	TL → OCB	0.071	0.107	0.509	Not Supported
2	LC → OCB	0.589	0.120	0.000	Supported
3	JS → OCB	0.220	0.107	0.040	Supported
4	TL → LC	0.772	0.049	0.000	Supported
5	TL → JS	0.812	0.037	0.000	Supported
6	TL→ LC → OCB	0.455	0.101	0.000	Supported
7	TL→ JS → OCB	0.179	0.089	0.046	Supported

**Notes:** TL, transformational leadership; LC, learning culture; JS, job satisfaction; OCB, organizational citizenship behavior.

**Figure 1:**
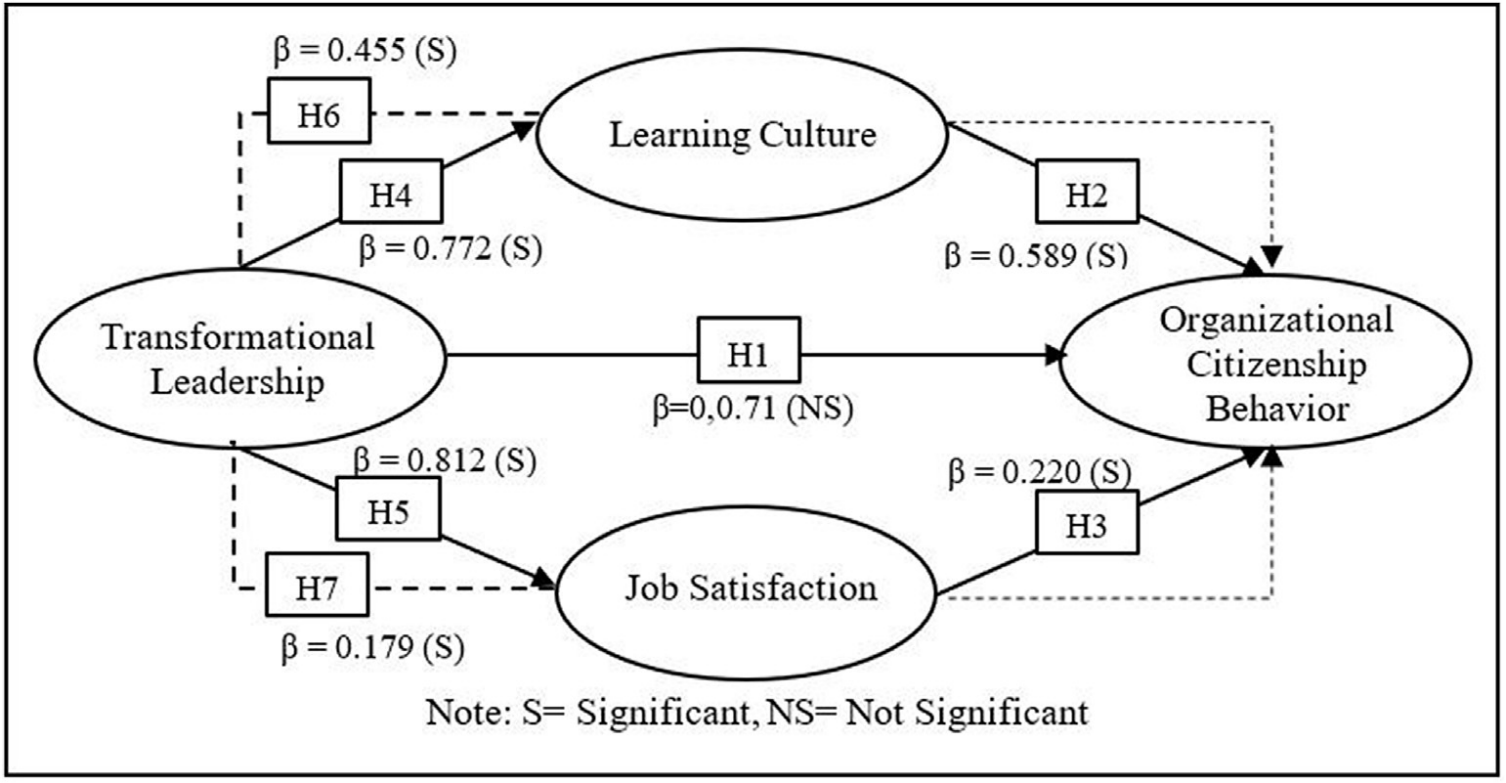
Diagram of hypotheses in PLS.

Based on the results of hypothesis testing (see table Table 3), the first hypothesis is not as predicted; TL has no direct effect on nurses' OCB (β = 0.071, sig. = 0.509). The finding declares that TL does not have a significant positive influence on nurses' OCB. Meanwhile, LC (β = 0.589, sig. = 0.000) and JS (β = 220, sig. = 0.040) showed a significant positive effect on nurses OCB. Thus, H2 and H3 are accepted. Furthermore, TL had positive significant effect on both LC (β = 0.772, sig. = 0.000) and JS (β = 0.812, sig. = 0.000). Thus, H4 and H5 are accepted. The results of the LC mediation test in the relationship between TL and nurses' OCB also showed a positive and significant role (β = 0.455, sig. = 0.000). Therefore, H6 is accepted. Also, JS was shown to have a positive and significant role in the relationship between TL and OCB (β = 0.179, sig. = 0.046). So H7 is supported. The following diagram (Figure 1) shows the path coefficients of the proposed hypothesis.

Previous studies tested the influence of TL on OCB showed positive and significant results (Jun, 2017; Mekpor and Dartey-Baah, 2017; Supriyanto et al., 2020). A study on 219 nurses in South Korea (Jun, 2017) verified that TL strongly influences the high OCB of nurses in hospitals. The finding, recently confirmed by Supriyanto et al. (2020), revealed that TL plays a key predictor of employees' voluntary work behaviors. Although most previous investigations showed significant results, the findings of this study indicate different results. TL, in this study, did not establish a substantial relationship with nurses' OCB. This is in line with the research results of Kim (2014), which states that TL directly does not show a significant relationship with OCB. The weak role of TL in improving nurses' OCB cannot be separated from their work characteristics, which are required to do extra work even though a leader does not provide encouragement and inspiration (Arifiani et al., 2016).

Although TL tends to have a less vital role in promoting OCB (Kim, 2014), The findings of this study prove that this leadership style shows a positive and significant signal on LC and JS. Froehlich et al. (2014) stated that employees' core skills and perceived career development resulting from LC were formed because of the leader's attention to each employee. A study on 324 employees in a Nigerian hospital also confirmed the findings of this study that idealized influence, inspirational motivation, intellectual stimulation, and individualized consideration as the main elements of TL strongly encourage JS (Ohunakin et al., 2019). Both JS and LC, in our study, showed a powerful influence on nurses' extra-role behavior. This also enriches the previous literature regarding the relationship between LC and OCB (Saoula et al., 2018) and between JS and OCB (Ng et al., 2019).

The present study also verifies the indirect relationship between TL and OCB through LC and JS. For LC, the leader as a central figure has a significant role in building organizational culture (Schein, 2010), including LC. Champoux (2011) stated that organizational culture through the learning process enables to shape people's behavior. Leaders who are active as participants in learning programs (role models) can increase the motivation of their members to do the same (Popper and Lipshitz, 2000). Other evidence suggests that inspiring leaders can encourage employees to engage in learning programs (Danish et al., 2014), which in turn motivates them to exhibit civic behavior (Saoula et al., 2018). Employees with great potential can show OCB when their organization strongly encourages them to be more involved in learning programs (Islam et al., 2016).

Finally, JS is also convincingly proven to be a mediator in the relationship between TL and OCB of nurses. Misra and Srivastava (2018) revealed that high employees' JS is closely related to TL's application. The greater the leader's role in motivating, addressing employees' personal needs, and performing ideally in the workplace, the more positive the contribution to the perception of employees' JS (Supriyanto et al., 2020). In the context of health sector organizations, TL is more acceptable than transactional leadership to increase nurse JS (Ahmad et al., 2013) and encourage discretionary behavior among them (Nasra and Heilbrunn, 2016). Hurst et al. (2017) and Biagioli et al. (2018) also stated that the more satisfied nurses are with their work, the more often they engage in voluntary work behaviors.

## 5 Conclusion

The present study explored the relations between TL, LC, JS, and OCB in Indonesian hospitals. LC and JS in this study were placed as mediation, which aims to find out more about the indirect effect of TL on OCB. The finding reveals that TL directly is not a pivotal factor in nurses' citizenship behavior in Indonesian hospitals. TL was only able to influence nurses' OCB when LC was placed as a mediator. This also applies to the JS mediation role. Therefore, hospital managers in Indonesia are advised to improve a systematic learning program by involving nurses as critical participants to encourage OCB among nurses. This is important, considering that nurses been actively engaged in the organizational learning agenda have high involvement in OCB. The high OCB of nurses was also verified by how well their level of satisfaction with their work was. The higher the nurses feel satisfied with their work, the higher their tendency to display OCB. Thus, it is indispensable for managers to pay more attention to nurses' JS, such as supporting them when conveying ideas or ideas, facilitating individual capacity development, and ensuring a comfortable working environment.

### 5.1 Limitations and further research

The present study, like most prior research, has several limitations requiring to be handled in future research. First, this study only considers non-leader nurses as research participants, allowing for bias in assessing statements, especially those related to self-assessment, such as JS and OCB. Further research is suggested to involve leaders in evaluating JS and OCB among nurses. Second, data collection is carried out at one moment, making it difficult to establish long-term trends. Future research is highly recommended to use longitudinal analysis, which allows data collection at different moments and times. Third, the present study is only concerned with the relationship between TL, LC, JS, and OCB, and TL shows no significant effect on OCB. Therefore, including the servant leadership variable may be more contextual with service organizations such as hospitals. In addition, to enrich the literature in this discipline, testing the effects of moderator variables should also be considered for future research.

## Declarations

### Author contribution statement

Idris Idris: Conceived and designed the experiments; Analyzed and interpreted the data; Wrote the paper.

Nanang AS.: Performed the experiments.

Budi Eko Soetjipto: Conceived and designed the experiments.

Achmad Sani Supriyanto: Contributed reagents, materials, analysis tools or data.

### Funding statement

This research did not receive any specific grant from funding agencies in the public, commercial, or not-for-profit sectors.

### Data availability statement

Data included in article/supplementary material/referenced in article.

### Declaration of interests statement

The authors declare no conflict of interest.

### Additional information

No additional information is available for this paper.

## References

[bib1] Ahmad A.R., Adi M.N.M., Md Noor H., Rahman A.G.A., Yushuang T. (2013). The influence of leadership style on job satisfaction among nurses. Asian Soc. Sci..

[bib2] Arifiani R.S., Astuti E.S., Ruhana I. (2016). The effect of transformational leadership on organizational citizenship behavior and job satisfaction (study on nurses of RSUD. Dr. Saiful Anwar malang). Jurnal Administrasi Bisnis (JAB).

[bib3] Bass, Bernard M., Avolio B.J. (2007). International journal of public administration transformational leadership and organizational culture. Int. J. Publ. Adm..

[bib4] Bass, Bernard M., Bass R. (2008).

[bib5] Bass B.M. (1985).

[bib7] Biagioli V., Prandi C., Nyatanga B., Fida R. (2018). The role of professional competency in influencing job satisfaction and organizational citizenship behavior among palliative care nurses. J. Hospice Palliat. Nurs..

[bib8] Boamah S.A., Spence Laschinger H.K., Wong C., Clarke S. (2018). Effect of transformational leadership on job satisfaction and patient safety outcomes. Nurs. Outlook.

[bib9] Champoux J.E. (2011).

[bib10] Danish R.Q., Munir Y., Ishaq M.I., Arshad A. (2014). Role of organizational learning, climate, and justice on teachers' extra-role performance. J. Basic Appl. Sci. Res..

[bib11] Egan T.M., Yang B., Bartlett K.R. (2004). The effects of organizational learning culture and job satisfaction on motivation to transfer learning and turnover intention. Hum. Resour. Dev. Q..

[bib12] Eisenberg A., Davidova J., Kokina I. (2018). The interrelation between organizational learning culture and organizational citizenship behavior. Rural Environ. Edu. Pers. (REEP).

[bib13] Fairbrother G., Jones A., Rivas K. (2010). Development and validation of the nursing workplace satisfaction questionnaire (NWSQ). Contemp. Nurse.

[bib14] Froehlich D., Segers M., Van den Bossche P. (2014). Informal workplace learning in Austrian banks: the influence of learning approach, leadership style, and organizational learning culture on managers' learning outcomes. Hum. Resour. Dev. Q..

[bib15] García-Morales V.J., Jiménez-Barrionuevo M.M., Gutiérrez-Gutiérrez L. (2012). Transformational leadership influence on organizational performance through organizational learning and innovation. J. Bus. Res..

[bib16] Garvin D.A. (1993). Building a learning organization. Harv. Bus. Rev..

[bib17] Gruneberg M.M. (1979).

[bib18] Gusrita G., Syahrizal S. (2020). Effect of affective commitment on organizational citizenship behavior and emotional performance with emotional intelligence as a moderation variable on nurse handling of COVID-19 RSUP. Adv. Econ. Bus. Manag. Res..

[bib19] Hair J.F., Page M., Brunsveld N. (2020).

[bib20] Hambali M., Idris I. (2020). Transformational leadership, organizational culture, quality assurance, and organizational performance: a case study in Islamic higher education institutions (IHEIS). J. Appl. Manag..

[bib21] Hartog D. N. Den, Muijen J. J. Van, Koopman P.L. (1997). Transactional versus transformational leadership: an analysis of the MLQ. J. Occup. Organ. Psychol..

[bib22] Hurst C.S., Baranik L.E., Clark S. (2017). Job content plateaus: justice, job satisfaction, and citizenship behavior. J. Career Dev..

[bib23] Idris I., Adi K.R. (2019). Transformational leadership and team performance: the role of innovation in Indonesia property agent industry. Adv. Econ. Bus. Manag. Res..

[bib24] Idris I., Adi K.R., Soetjipto B.E., Supriyanto A.S. (2020). The mediating role of job satisfaction on compensation, work environment, and employee performance: evidence from Indonesia. Enterpren. Sustain. Issues.

[bib25] Idris, Setiawan M., Susilowati C., Supriyanto A.S., Ekowati V.M., Muhammad F. (2020). Examining the role of political skill in transformational leadership and organizational performance; Empirical study from Indonesia. SMART J. Bus. Manag. Stud..

[bib26] Islam T., Khan M.M., Bukhari F.H. (2016). The role of organizational learning culture and psychological empowerment in reducing turnover intention and enhancing citizenship behavior. Learn. Organ..

[bib27] Jo S.J., Joo B.K. (2011). Knowledge sharing: the influences of learning organization culture, organizational commitment, and organizational citizenship behaviors. J. Leader. Organ Stud..

[bib28] Jun S.Y. (2017). Mediating effect of social capital between transformational leadership and organizational commitment of nurses in hospitals. J. Korean Acad. Nurs. Adm..

[bib29] Katz D. (1964). The motivational basis of organizational behavior. Behav. Sci..

[bib30] Katz D., Kahn R.L. (1966).

[bib31] Khalili A. (2017). Transformational leadership and organizational citizenship behavior. Leader. Organ. Dev. J..

[bib32] Kim H. (2014). Transformational leadership, organizational clan culture, organizational affective commitment, and organizational citizenship behavior: a case of South Korea's public sector. Publ. Organ. Rev..

[bib33] Lee S.E., MacPhee M., Dahinten V.S. (2020). Factors related to perioperative nurses' job satisfaction and intention to leave. Jpn. J. Nurs. Sci..

[bib34] Luthans F. (2011).

[bib35] Margerison C. (2001). Team performance management: an international journal team competencies. Int. J. Iss. Team Perform. Manag.: Int. J..

[bib36] Marsick V.J. (2013). The dimensions of a learning organization questionnaire (DLOQ): introduction to the special issue examining DLOQ use over a decade. Adv. Develop. Hum. Resour..

[bib37] Marsick V.J., Watkins K.E. (2003). Demonstrating the value of an organization's learning culture: the dimensions of the learning organization questionnaire. Adv. Develop. Hum. Resour..

[bib38] Mekpor B., Dartey-Baah K. (2017). Leadership styles and employees' voluntary work behaviors in the Ghanaian banking sector. Leader. Organ. Dev. J..

[bib39] Misra S., Srivastava K.B.L. (2018). Team-building competencies, personal effectiveness, and job satisfaction: the mediating effect of transformational leadership and technology. Manag. Labour Stud..

[bib40] Monroe C., Loresto F., Horton-Deutsch S., Kleiner C., Eron K., Varney R., Grimm S. (2021). The value of intentional self-care practices: the effects of mindfulness on improving job satisfaction, teamwork, and workplace environments. Arch. Psychiatr. Nurs..

[bib41] Na-Nan K., Kanthong S., Joungtrakul J., Smith I.D. (2020). Mediating effects of job satisfaction and organizational commitment between problems with performance appraisal and organizational citizenship behavior. J. Open Innov.: Technol. Market Complex..

[bib42] Narzary G., Palo S. (2020). Structural empowerment and organizational citizenship behavior: the mediating–moderating effect of job satisfaction. Person. Rev..

[bib43] Nasra M.A., Heilbrunn S. (2016). Transformational leadership and organizational citizenship behavior in the Arab educational system in Israel: the impact of trust and job satisfaction. Educ. Manag. Adm. Leader.

[bib44] Ng L.P., Choong Y.O., Kuar L.S., Tan C.E., Teoh S.Y. (2019). Job satisfaction and organizational citizenship behavior amongst health professionals: the mediating role of work engagement. Int. J. Healthc. Manag..

[bib45] Nguni S., Sleegers P., Denessen E. (2006). Transformational and transactional leadership effects on teachers' job satisfaction, organizational commitment, and organizational citizenship behavior in primary schools: the Tanzanian case. Sch. Effect. Sch. Improv..

[bib46] Ocampo L., Acedillo V., Bacunador A.M., Balo C.C., Lagdameo Y.J., Tupa N.S. (2018). A historical review of the development of organizational citizenship behavior (OCB) and its implications for the twenty-first century. Person. Rev..

[bib47] Ohunakin F., Adeniji A.A., Oludayo O.A., Osibanjo A.O., Oduyoye O.O. (2019). Employees' retention in Nigeria's hospitality industry: the role of transformational leadership style and job satisfaction. J. Hum. Resour. Hospit. Tourism.

[bib48] Organ D.W., Podsakoff P.M., MacKenzie S.B., Podsakoff N.P. (2016). The Oxford Handbook of Organizational Citizenship Behavior.

[bib49] Park S., Kim E. (2018). Fostering organizational learning through leadership and knowledge sharing. J. Knowl. Manag..

[bib50] Podsakoff M.P., MacKenzie S.B., Paine B.J., Bachhrach D.G. (2000). Organizational citizenship behaviors: a critical review of the theoretical and empirical literature and suggestions for future research. J. Manag..

[bib51] Podsakoff P.M., Mackenzie S.B., Moorman R.H., Fetter R. (1990). Transformational leader behavior and their effects on followers' trust in leader, satisfaction, and organizational citizenship behaviors. Leader. Q..

[bib52] Popper M., Lipshitz R. (2000). Organizational learning: mechanisms, culture and feasibility. Manag. Learn..

[bib53] Robbins S., Judge T.A. (2017). *Fortune* (17 Global).

[bib54] Sani A., Ekowati V.M., Wekke I.S., Idris I. (2018). Respective contribution of entrepreneurial leadership through organizational citizenship behavior in creating employees performance. Acad. Enterpren. J..

[bib55] Saoula O., Johari H., Fareed M. (2018). A conceptualization of the role of organizational learning culture and organizational citizenship behavior in reducing turnover intention. J. Bus. Retail Manag. Res..

[bib56] Schein E.H. (2010).

[bib57] Smith C.A., Organ D.W., Near J.P. (1983). Organizational citizenship behavior: its nature and antecedents. J. Appl. Psychol..

[bib58] Supriyanto A.S., Ekowati V.M., Idris I., Iswanto B. (2020). Leadership styles as a predictor of the voluntary work behaviors of bank employees. Int. J. Econ. Manag..

[bib59] Vermeir P., Blot S., Degroote S., Vandijck D., Mariman A., Vanacker T., Peleman R. (2018). Intensive & Critical Care Nursing Communication satisfaction and job satisfaction among critical care nurses and their impact on burnout and intention to leave: a questionnaire study. Intensive Crit. Care Nurs..

[bib60] Zurahmi D., Masdupi E., Patrisia D. (2019). The effect of transformational leadership, quality of work life (QWL) on organizational citizenship behavior (OCB) at tapan regional general hospital. Adv. Econ. Bus. Manag. Res..

